# Deaf awareness workshop for medical students – an evaluation

**DOI:** 10.3205/zma001514

**Published:** 2021-11-15

**Authors:** Janina Kruse, Anja Zimmermann, Michael Fuchs, Daisy Rotzoll

**Affiliations:** 1 Leipzig University, Medical Faculty, Leipzig, Germany; 2 Leipzig University, Medical Faculty, Skills and Simulation Centre, LernKlinik, Leipzig, Germany; 3 Leipzig University, Medical Faculty, Department of Otorhinolaryngology, Section of Phoniatrics and Audiology, Leipzig, Germany

**Keywords:** medical students, deaf awareness, deafness, knowledge, competence

## Abstract

**Background: **Due to a lack of communication strategies and knowledge about the Deaf community, health care professionals are often not prepared to provide deaf or hard of hearing patients with accessible and adapted healthcare.

**Methods**: In the present study, a workshop was designed to determine the effect of deaf awareness training on medical students concerning their gain of knowledge regarding deafness and their competence in providing adapted communication and healthcare for deaf and hard of hearing patients. 95 medical students were evaluated in an online survey prior to as well as following the workshop. The workshop was held online in three consecutive sessions.

**Results:** Students reported a substantially more confident approach to working with hearing impaired patients and indicated that an online learning environment is a suitable and helpful alternative to face-to-face teaching. Participants improved significantly in all evaluated items concerning their knowledge and competence (p<0.001). Furthermore, measurements revealed interaction effects between students’ current period of study and the point in time of self-evaluation before and after the workshop. Preclinical students not only catch up but even surpass their clinical peers concerning their learning outcome.

**Conclusion: **Reviewing the results obtained by this study, we are optimistic with respect to all participants’ highly positive experiences and learning outcomes. Deaf awareness training should be included in the curriculum of all medical faculties.

## Zusammenfassung

**Hintergrund: **Aufgrund mangelnder Kommunikationsstrategien und eines Mangels an Wissen ist medizinisches Fachpersonal häufig nicht gut darauf vorbereitet, gehörlosen oder schwerhörigen Patient*innen einen adäquaten Zugang zur Gesundheitsversorgung zu ermöglichen.

**Methoden:** In der vorliegenden Studie wurde ein Workshop konzipiert, um die Wirkung eines Gehörlosen-Sensibilisierungstrainings für Medizinstudierende auf deren Wissenszuwachs hinsichtlich Gehörlosigkeit und ihrer Kompetenz in der Gesundheitsversorgung eine angepasste Kommunikation für Patient*innen mit Hörbeeinträchtigung bereitzustellen, zu untersuchen. 95 Medizinstudierende wurden sowohl vor als auch nach dem Workshop mittels einer Online-Umfrage befragt. Der Workshop wurde online in drei aufeinanderfolgenden Sitzungen abgehalten.

**Ergebnisse: **Die Studierenden berichteten einen Zugewinn an Selbstbewusstsein im Umgang mit hörgeschädigten Patient*innen und bewerteten die digitale Lernumgebung als geeignete und hilfreiche Alternative zum Lernen in Präsenz. Die Teilnehmenden verbesserten sich signifikant in allen Kompetenz- und Wissensitems (p<0,001). Darüber hinaus zeigten sich Interaktionseffekte zwischen dem aktuellen Studiensemester und dem Zeitpunkt der Selbsteinschätzung vor bzw. nach dem Workshop. Die vorklinischen Studierenden holten nicht nur auf, sondern übertrafen ihre Kommilitonen der klinischen Semester hinsichtlich ihrer Lernergebnisse.

**Fazit:** Die Ergebnisse dieser Studie zeigen äußerst positive Erfahrungen und Lernerfolge der Teilnehmenden. Ein Training für Studierende zur Sensibilisierung für den Umgang mit tauben und schwerhörigen Patient*innen sollte in die Lehrpläne aller medizinischer Fakultäten aufgenommen werden.

## Background

In Germany, approximately one in five people is affected by hearing loss, equating to about 16 million people. Around 140,000 depend on a sign language interpreter and 80,000 are deaf [1]. Studies have revealed that healthcare professionals often are not prepared to provide deaf or hard of hearing patients with accessible and adapted healthcare [2], [3]. These patients feel that “most physicians, largely unconsciously, hold fundamental assumptions about deafness” ([4] p. 357) and “some physicians do not adequately respect patients’ intelligence, motivation, and desire to understand and participate in their healthcare” ([4] p. 357) which leads to a lack of respect for the Deaf community *(“Deaf” describes culturally Deaf members of the Deaf community, while “deaf” simply refers to the medical condition of hearing loss)* that sees itself rather as a linguistic minority than as having a disability [5]. Communication barriers as well as the insufficient knowledge about Deaf culture can interfere with building a trusting relationship [6] and cause fear, mistrust, and frustration [7]. Thus, deaf and hard of hearing patients might not be satisfied with their healthcare [8] and seek physicians more often [9], [10].

There are a number of barriers [11] preventing deaf patients from having equal access to medical treatment. Many physicians require their deaf patients to speechread, yet only about 30% [12], [13], [14] of spoken words can accurately be speechread. This is widely overestimated by physicians leading to misunderstandings [12]. Moreover, German can be considered a second language after sign language acquisition in childhood [15]. The average prelingual deaf person reads and writes at a fourth grade level [15], [16]. Thus, deaf patients often exhibit lower health literacy than do hearing patients and therefore require adapted communication to ensure reliable transmission of health-relevant information [2], [17].

In 2009, the German government ratified the UN Convention on the Rights of Persons with Disabilities, clearly stating that people with disabilities must be provided with accessible healthcare through training [https://www.behindertenrechtskonvention.info/] of healthcare professionals. Since 2015, the National Competence-Based Learning Objectives for Undergraduate Medical Education (NKLM) have offered a detailed description of the skillset a medical student in Germany should be provided with. Concerning the treatment of deaf and hard of hearing patients, the NKLM requires future physicians to adapt the medical setting in order to meet patients’ specific needs [http://www.nklm.de], [18]. Furthermore, it states that medical students should be able to reflect on the use of (non)professional interpreters, work competently with them and be aware of the physician’s role in an interpreter’s presence [http://www.nklm.de], [18]. 

In prior research, various programs and workshops have focused on medical students’ attitudes, perception of deafness, cultural competence, and knowledge [19], [20], [21], [22], [23]. These studies have revealed that medical students acquire a more positive attitude to deaf individuals and higher knowledge scores, as well as confidence in working with deaf patients [19], [20], [21], [22], [23]. In the present study, we offered a one-off, extracurricular deaf awareness workshop for medical students to meet both sets of demands established in the NKLM. As the first of its kind in Germany known to the authors, the objective is to evaluate the effectiveness of an online deaf awareness workshop regarding medical student’s knowledge of and competence in communication with deaf and hard of hearing patients in a medical setting and to increase cross-cultural competence in future health professionals. This can facilitate deaf and hard of hearing patients’ access to health information and enhance adapted healthcare [19]. 

## Methods

### Context

As part of the program *Breaking the Silence*, a workshop was designed to teach medical students in communication with deaf and hard of hearing patients. Established in 2013, *Breaking the Silence* is a student initiative aiming for a better understanding of Deaf culture in future doctors. It is one of many programs of the *German Medical Students’ Association* (bvmd), globally represented by the *International Federation of Medical Students’ Associations* (IFMSA). A pre-post study design was chosen to allow for an evaluation prior to and following the intervention to analyze the immediate impact of the workshop on student learning.

#### Participant recruitment

Participants’ recruitment took place via the nationwide network of the BVMD in close exchange with student councils at local medical faculties. The sample comprised 130 workshop participants, of which 100 respondents completed both surveys (77%) before and after the workshop. Five were omitted as they had graduated in the meantime, were trained audiologists or students from disciplines other than medicine, leaving the remaining sample with 95 respondents.

#### Workshop

**Figure 1:**
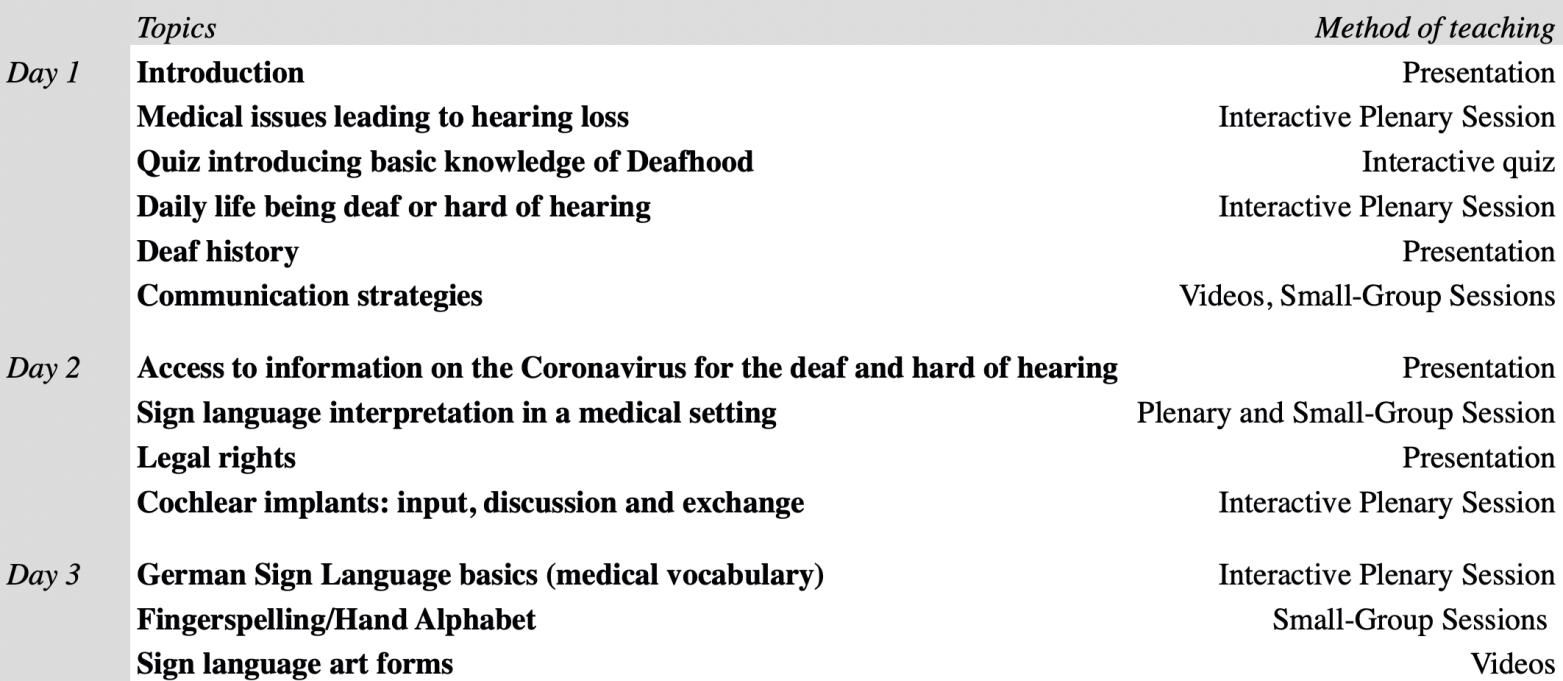
Workshop “Breaking the Silence” – topics covered in tow-hour online sessions

The workshop was planned as an extra-curricular face-to-face seminar at multiple universities across Germany. Due to the COVID-19 pandemic it was decided to switch to an online format. Using a video conference tool, participants were able to interact directly, work on certain tasks in small groups and learn the very basics of German Sign Language. As can be seen in figure 1 (Fig. 1), the workshop was split into three individual sessions of two hours over one week, being held three times in April 2020. Participants were expected to spend 30-60 minutes on individual preparatory work prior to each session using provided material. Each session was carried out by a group of three to four medical students with experience in conducting deaf awareness workshops. They all have been contributing to *Breaking the Silence* for several years and were trained within the program. Allowing participants to develop basic skills in signing, a deaf sign language teacher was present for the third session.

#### Survey

Before and after taking part in the workshop, participants were asked to fill out an online questionnaire encouraging anonymous answers after providing informed consent about statistical analysis of the shared data. The pre-survey included questions concerning participants’ demographic information and motivation to attend the workshop, prior contact to deaf or hard of hearing people, knowledge of Deaf culture and communication, as well as a short assessment of the skillset needed to efficiently treat deaf or hard of hearing patients in a medical context. The post-survey reassessed participants’ knowledge and skillset besides presenting a set of questions evaluating the workshop and online learning environment. The survey questions were developed based on a review of the literature [4], [11], [24], [25] and were rated on a 6-point Likert-scale (1=strongly disagree, 6=strongly agree). Open-ended questions in the post-survey encouraged participants to reflect on their online learning experience and identify their most important findings.

#### Data analysis

With consent from the Medical Faculty’s ethics committee (case number 521/19-ek) data analyses were performed using SPSS, version 26. Answers of both surveys were matched by an anonymous code provided by the respondents. To determine significant differences in participants’ view on the workshop’s relevance for medical students before and after the workshop, a one-factorial ANOVA was used. To compare the main effects of time (as in pre- and post-workshop) and period of study (as in preclinical or clinical study period), as well as the interaction effect between time and period of study on knowledge and competence in workshop attendees, a two-factorial repeated measurement was conducted. A p-value less than 0.05 was considered to be statistically significant. The evaluation of the open-ended questions in report form is carried out as a supplement to the quantitative analysis.

## Results

A total of 95 participants, aged 18 to 33 (mean=23.26, SD=2.98), completed the questionnaires at both evaluation points. Two-thirds (65.26%) of the students were currently enrolled in a clinical semester. Most of these students (65.3%) had never been in contact with a deaf or hard of hearing person before. 90.5% of the participants were female, while the nationwide gender distribution also shows an imbalance with 61.5% female medical students [https://de.statista.com/statistik/daten/studie/200758/umfrage/entwicklung-der-anzahl-der-medizinstudenten/]. Students studied at 19 different medical faculties with larger groups based in Halle, Tübingen, and Münster (n=14, n=16, n=13 respectively). Of those who had previously been in contact with a deaf or hard of hearing person (n=53), most interactions took place in a medical setting (n=33). 

Overall, the workshop was rated 1.4 according to the German school grading system, ranging from 1 (excellent) to 6 (insufficient). Students found the topics treated exceedingly helpful from a personal (82.1%≥5p) and a professional point of view (84.2%≥5p). They reported having a substantially more confident approach to hearing impaired patients (77.9%≥5p) along with a personal benefit (88.4%≥5p) from having taken part in deaf awareness training.

### Knowledge and competence

**Figure 2:**
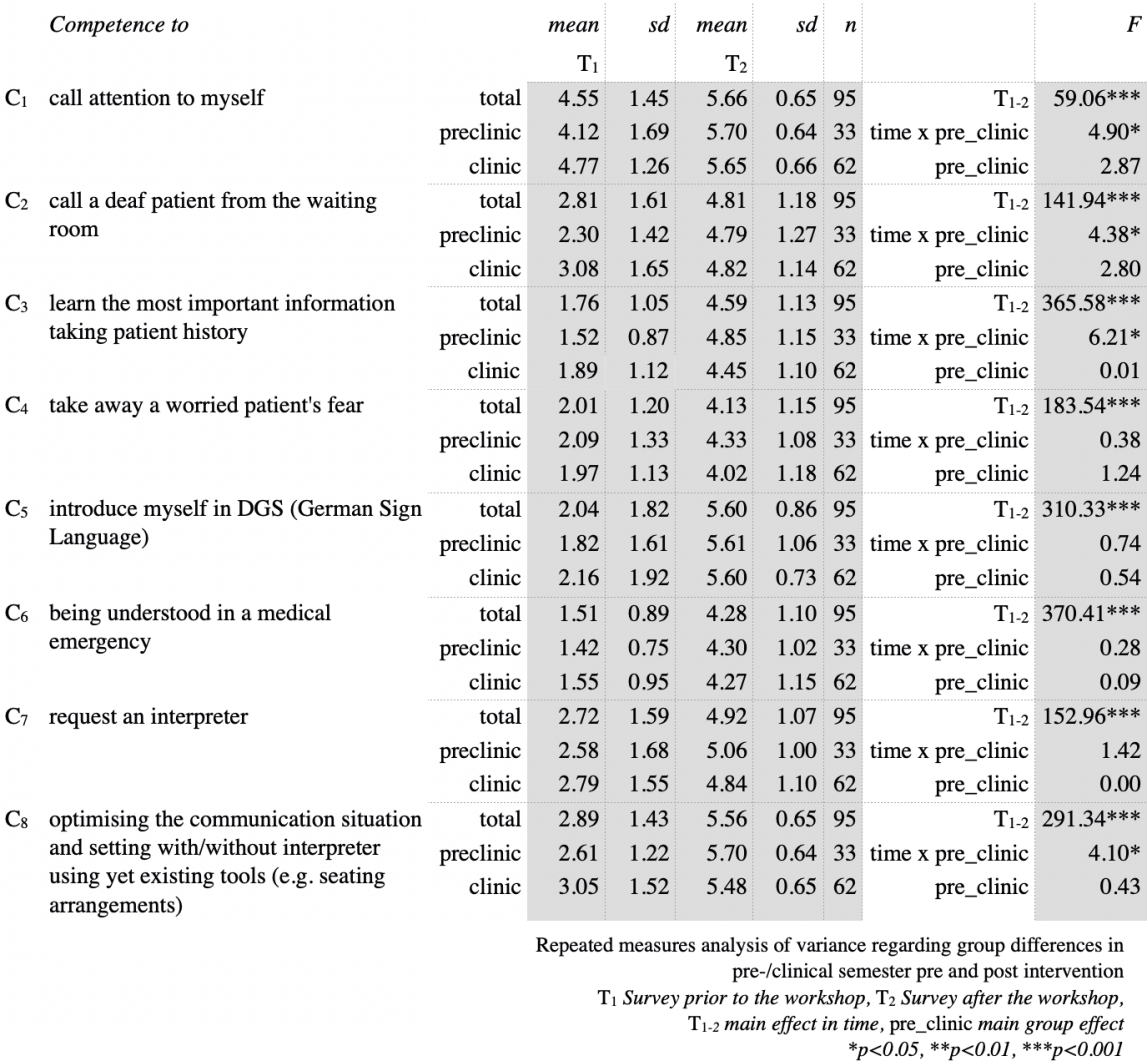
Survey results reviewing competence in participants before and after the workshop

**Figure 3:**
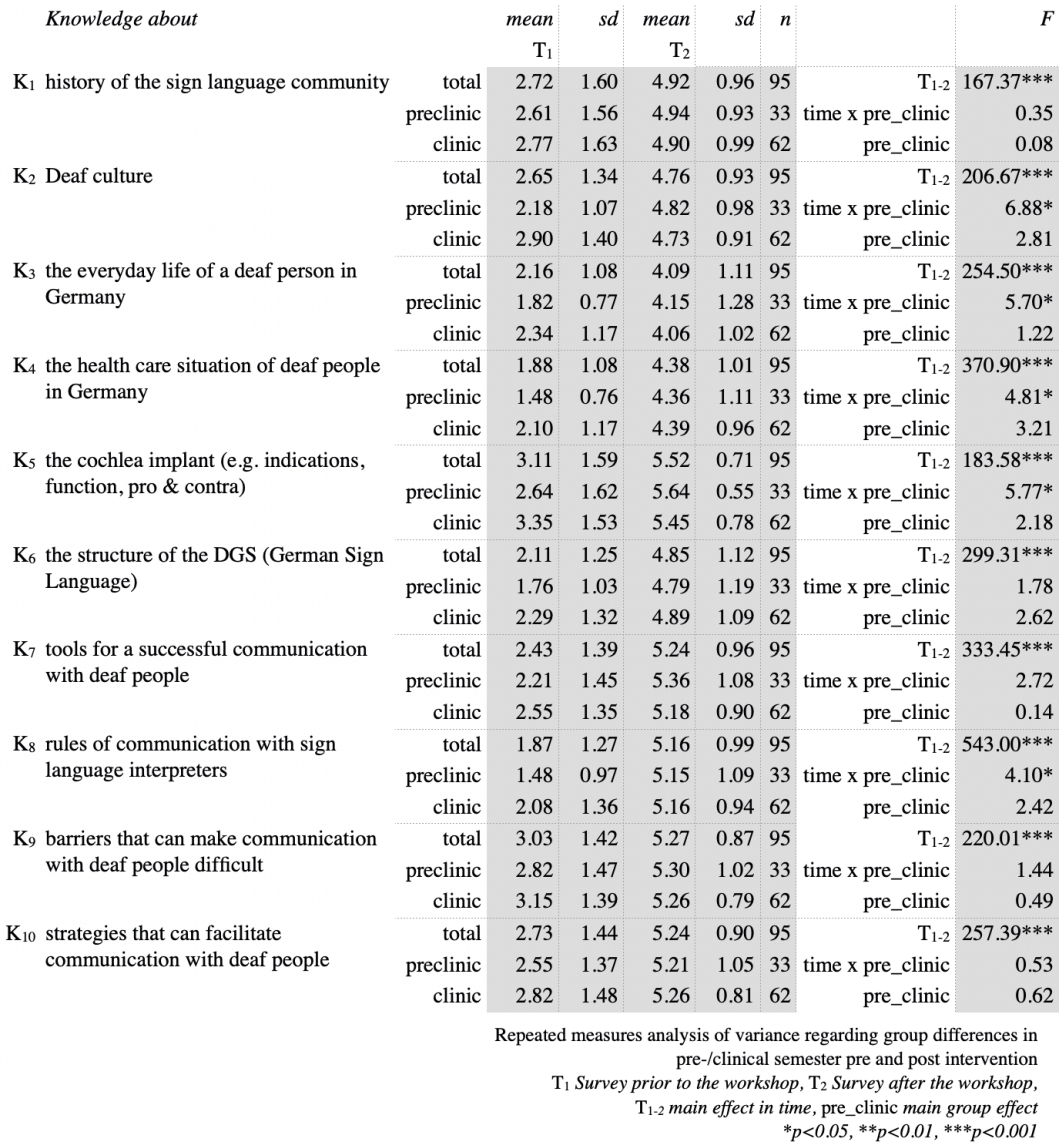
Survey results reviewing knowledge in participants before and after the workshop

The main focus of this study has been students’ increase in competence towards and knowledge about deaf and hard of hearing patients. As can be seen in figure 2 (Fig. 2) and figure 3 (Fig. 3), prior to the workshop overall knowledge concerning deafness and the competence in treating deaf patients was very low. Comparing students’ self-evaluation regarding knowledge about the Deaf community and their competence in interacting with deaf or hard of hearing patients, the most striking result is that in all of the evaluated items students improved significantly (p<0.001). Considering the items C_1–3,8_ and K_2–5,8_ there were interactions found between students’ current period of study and the point in time of self-evaluation before and after the workshop. Exhibiting far less knowledge and competence towards deaf and hard of hearing patients than do clinical students prior to the workshop, preclinical students not only catch up but even surpass their clinical peers concerning the learning outcome in these items.

Prior to the workshop, students found the general topic of deaf and hard of hearing patients to be very important (92.6%≥5p) and relevant for their careers (74.7%=5p), but they were not entirely convinced of the topic’s relevance for medical students (62.1%≥5p). After the workshop, even more students found this subject important personally (94.7%≥5p), also with respect to their medical careers (84.2%≥5p). Furthermore, a significant increase was revealed (mean T1=4.81, SD T1=1.22, mean T2=5.06, SD T2=1.19, F=5.94, p=0.02) concerning the participants’ views on the topic’s relevance for all medical students (73.7%≥5p). 

#### eLearning

Despite the online workshop being a new way of learning for the far most (75.8%≥5p), students indicated it to be a suitable and helpful alternative to face-to-face teaching (81.1%≥5p). Participants reported having worked in depth through the given preparatory material and found them very helpful (83.7%≥5p). The individual two-hour online sessions were found to be highly appropriate in terms of time and content. Only very few students reported minor technical difficulties, due to unstable internet connection in their work environment at home.

#### Open questions

In the end of the post-workshop survey, open questions provided attendees with the opportunity to report their experiences in an online teaching environment and display their three key learnings. Many students were surprised by their immense increase in knowledge gained during a fairly compact workshop. Barely knowing anything about deafness prior to the workshop they even acquired basic communication skills. Moreover, some students emphasized the importance of touching this topic during medical training and a great number of respondents stated a loss of fear to communicate with deaf or hard of hearing patients. Some were even looking forward to getting in touch with a signing patient in the future.

Concerning the eLearning environment almost all respondents valued the very comfortable possibilities to take part in the workshop. No time got lost in travel and one could even fit the workshop in the personal daily routine. However, certain students mentioned being more easily distracted by their home environment. In some cases, a bad internet connection made it difficult to follow presentations. Participants noticed particular group dynamics enabled by the given anonymity and some students stated the lack of getting in touch with new people and valuable social group interactions.

## Discussion

For most of the 95 participants, the workshop presented an opportunity to familiarize themselves with topics on hearing loss and deafness for the first time. Some admitted to not having even realized the importance of deaf awareness training, before thinking in depth about communication barriers and patient treatment. Reviewing the study’s results regarding personal as well as professional benefits and the confidence gained by students, this theoretical training can be regarded enjoyable and highly effective.

This study focused on encouraging students’ knowledge and competence. Moreover, literature indicates the significant changes in students’ attitudes to deafness attending deaf awareness training [20]. However, students’ learning outcomes go far beyond, and allow students to transfer what they have learned about treating deaf or hard of hearing patients to other disability groups. Challenging perspectives for the first time and learning about the complexities for a disabled patient navigating healthcare, deepens a medical student’s perception of a patient’s experience and reduces health disparities [19]. This leads our students to become more aware and to be prepared to adequately treat all patients as future physicians.

### Differences in pre-/clinical students

Preclinical students exhibit far less knowledge and competence towards deaf and hard of hearing patients than do their peers in clinical semesters. Comparing these groups of students before attending the workshop, significant interactions can be seen in the items C_1–3,8_ and K_2–5,8_. Strikingly, these differences are no longer present after the workshop. It is thus reasonable to conclude that preclinical students benefit even more from deaf awareness training than do clinical students. The advantage of already having had first contact to patients, does not help clinical students profit more from deaf awareness training. Consequently, teaching future medical professionals in treating deaf or hard of hearing patients can be applied at any point in time during medical studies. However, integrating it in the undergraduate curriculum makes it possible to encounter this topic repeatedly in later years of study, and thus promotes a sustainable increase in learning.

#### Evaluation

Another promising finding was the seminars’ overall rating. Students were exceedingly satisfied and rated the workshop with a 1.4 according to the German school grading system in terms of not only content and preparatory material, but also prior communication with participants, atmosphere during the online group sessions, and students’ learning experience. While commenting on some concerns about lack of social interaction due to the eLearning format, participants very much valued the online learning opportunity. Comments suggest while interest in the deaf community remains a niche in the medical field, ensuring an easy access to participation definitely broadens the workshops’ audience. Nevertheless, we cannot determine whether our sample was composed of those students generally lacking knowledge in deafness or rather those who already had an interest in this field.

#### Implementation in medical curriculum

Workshop participants did not indicate even basic knowledge of how to successfully communicate with deaf and hard of hearing patients before having completed the workshop, although the most basic content addressed in the program should already have been included in their curriculum, since the NKLM demands a broad skillset in handling patients with a variety of disabilities [http://www.nklm.de]. Students’ extremely positive feedback and significant increase in expertise demonstrate how this crucial issue can be sufficiently covered even with limited resources and time. This makes it easily possible for all medical faculties to integrate deaf awareness training into their present curriculum. Moreover, the results highlight the importance of deaf awareness training for the students themselves. After the workshop, participants’ view on the importance of this type of training changed significantly. While respondents found the topic more interesting and relevant, they reached the conclusion that all medical students should be provided with such training. Viewing these results, it might be interesting to invite students from other medical professions to participate in the workshop ensuring adapted healthcare from all possible angles.

#### Previous contact with deafness

Approximately half of the participants stated they had had prior contact with a hearing-impaired person. Two thirds of this contact generally occurred in a medical setting and in one third of the cases, the contact happened specifically with patients being deaf or hard of hearing. On the one hand, it is reasonable to assume that students who were previously in touch with deafness would be more familiar with their communication needs and therefore have an advantage over students without prior exposure. On the other hand, we did not specify the kind of contact when asking students about their prior encounters with deaf or hard of hearing patients. Intentionally leaving this open to interpretation, indication of all types of contact was ensured. Yet, Cooper et al. (2003) [26] state that it is rather the frequency of contact with deaf or hard of hearing people of equal or higher status that relate to more positive attitudes. It would thus be interesting to determine precisely which kind of contact had occurred and what impact it had on students’ deaf awareness and communication skills. However, consideration must be given to whether simple exposure to deaf and hard of hearing patients during daily routines in medical facilities will equip future healthcare professionals with sufficient understanding for deaf culture and expertise, or if specialized deaf awareness training for healthcare workers is a necessity. This also allows interdisciplinary training for doctors, nurses, therapists, and other medical professionals to be carried out and should be addressed in future research. 

#### Limitations

We evaluated students’ knowledge and competence immediately before and after the workshop. Future research should consider the potential effects of students’ benefit from deaf awareness training using longitudinal data in order to assess the sustainability of acquired competences. Moreover, our sample of students was 90.5% female. A gender balance should be respected in future research to make results applicable to all student groups. Being an elective workshop only participants were involved that had a certain baseline of motivation. Results may vary when participation is compulsory. 

Another limitation includes the shortcoming of direct interaction due to the online format. Despite most of the participants joining the online sessions with their camera activated, it was not possible to verify who was continuously present and who might have been distracted occasionally. Further, we could not provide the students with a personal experience of communicating with a deaf person or a sign language interpreter. However, applying theoretical knowledge can be considered a crucial element of long-term learning. While our deaf sign language teacher offered a short introduction to signing, this was a different experience compared to how a direct interaction would have been. Working with a computer screen can be challenging in the process of language acquisition since signing depends on three-dimensional aspects of movements. Taking all these factors into consideration, a face-to-face workshop has to be seen as the preferred method.

In further research it might be interesting to evaluate participants’ learning outcome employing a practical exam featuring conducted interviews with deaf simulated patients. Due to the online format of our workshop, this kind of examination process was not applicable.

## Conclusion

Effort is still required to equip all future physicians not only with awareness of deaf patients’ communication needs, but also the competence necessary to address their patients with an individual approach and to equally involve deaf and hard of hearing patients in the process of shared decision-making on their health. Inclusion of deaf awareness training in the undergraduate medical curriculum is therefore indispensable and should strongly be considered by all medical faculties. Reviewing the results achieved with this study, we are optimistic with respect to all participants’ highly positive experiences and learning outcomes, that even allow them to transfer what they have learned about treating deaf or hard of hearing patients to other disability groups. Although this study can only be understood as one of the steps necessary towards accessible healthcare for all patients, it has been very helpful to make use of the opportunity to foster the reduction of communication barriers and to broaden future medical professionals’ perspectives regarding the community of deaf and hard of hearing people.

## Acknowledgements

The authors would like to express their deepest appreciation to all members of the project *Breaking the Silence* and thank the participants of the survey for their contribution to improving medical teaching.

## Competing interests

The authors declare that they have no competing interests.
